# MCPH1: a window into brain development and evolution

**DOI:** 10.3389/fncel.2015.00092

**Published:** 2015-03-27

**Authors:** Jeremy N. Pulvers, Nathalie Journiac, Yoko Arai, Jeannette Nardelli

**Affiliations:** ^1^Sydney Medical Program, University of SydneySydney, Australia; ^2^U1141 InsermParis, France; ^3^Université Paris Diderot, Sorbonne Paris Cité, UMRS 1141Paris, France; ^4^Institut Jacques Monod, CNRS UMR 7592, Université Paris Diderot, Sorbonne Paris CitéParis, France

**Keywords:** MCPH1, microcephaly, brain development, brain evolution, mouse models, human

## Abstract

The development of the mammalian cerebral cortex involves a series of mechanisms: from patterning, progenitor cell proliferation and differentiation, to neuronal migration. Many factors influence the development of the cerebral cortex to its normal size and neuronal composition. Of these, the mechanisms that influence the proliferation and differentiation of neural progenitor cells are of particular interest, as they may have the greatest consequence on brain size, not only during development but also in evolution. In this context, causative genes of human autosomal recessive primary microcephaly, such as *ASPM* and *MCPH1*, are attractive candidates, as many of them show positive selection during primate evolution. *MCPH1* causes microcephaly in mice and humans and is involved in a diverse array of molecular functions beyond brain development, including DNA repair and chromosome condensation. Positive selection of *MCPH1* in the primate lineage has led to much insight and discussion of its role in brain size evolution. In this review, we will present an overview of *MCPH1* from these multiple angles, and whilst its specific role in brain size regulation during development and evolution remain elusive, the pieces of the puzzle will be discussed with the aim of putting together the full picture of this fascinating gene.

## Introduction: *MCPH1* in Brain Development and Evolution

The study of mammalian neurogenesis and cortical development stands at a fascinating intersection between neuroscience, cell biology, developmental biology, genetics, and evolutionary biology (Molnár et al., 2014; Paridaen and Huttner, 2014; Sun and Hevner, 2014). The studies of the genes that cause autosomal recessive primary microcephaly (MCPH) are exemplary of this exciting synthesis of research fields (Woods et al., 2005; Kaindl et al., 2010; Gilmore and Walsh, 2013). One of the causative genes of this condition, *MCPH1* (syn. *BRIT1*, *Microcephalin*), plays a role in brain development (Jackson et al., 1998, 2002), DNA damage repair (Xu et al., 2004; Lin et al., 2005; Peng et al., 2009), chromosome condensation (Neitzel et al., 2002; Trimborn et al., 2004; Yamashita et al., 2011), cancer (Chaplet et al., 2006; Rai et al., 2006; Richardson et al., 2011), germline function (Liang et al., 2010), and has also provided insights into brain evolution (Evans et al., 2004, 2005; Wang and Su, 2004; Ponting and Jackson, 2005). Many unanswered questions remain on this multifaceted gene, such as how the lack of *MCPH1* leads to microcephaly, its molecular mechanisms in neurogenesis, and the key question of its role in the evolution of brain size.

The development of the cerebral cortex begins with formation and patterning of the neural tube (Lumsden and Krumlauf, 1996; Rubenstein et al., 1998; Copp et al., 2003), which is followed by the amplification of neuroepithelial cells, the primary neural progenitor cells, and their subsequent differentiation into downstream progenitors and neurons, or “neurogenesis” (Götz and Huttner, 2005; Paridaen and Huttner, 2014; Sun and Hevner, 2014). A constellation of processes follows to form a fully developed cerebral cortex, including neuronal migration (Sidman and Rakic, 1973; Nadarajah and Parnavelas, 2002; Marín and Rubenstein, 2003), axon guidance (Tessier-Lavigne and Goodman, 1996; Dickson, 2002) and synaptogenesis (Garner et al., 2002; Waites et al., 2005). In the context of brain development and evolution, the embryonic development of the mammalian cerebral cortex (neocortex) is the subject of prime interest, being the seat of higher brain functions, and has powerful implications for primate and human evolution (Rakic, 2009; Clowry et al., 2010).

Investigations into cortical malformations give profound insight into not only developmental and molecular mechanisms, but also provide a platform to investigate the evolution of brain size and function (Walsh, 1999; Mochida and Walsh, 2001; Sun and Hevner, 2014). Amongst these conditions, congenital microcephaly of genetic etiology is of particular interest, as they allow the dissection of fundamental molecular and developmental mechanisms. Interestingly, these mechanisms may be affected in congenital microcephaly linked to environmental intrauterine insults, such as viral infections (Cheeran et al., 2009), alcohol, or other extrinsic cues, exemplified by the finding that *Mcph1*, the mouse ortholog of human *MCPH1*, was shown to be down-regulated in a mouse model of microcephaly induced by early embryonic exposure to a VIP (vasoactive intestinal peptide) antagonist (Passemard et al., 2011). *MCPH1* may be a common denominator in the pathway causing microcephaly, encompassing the spectrum of both environmental and genetic forms of microcephaly. Therefore, given its implication in diverse molecular and cellular mechanisms during brain development, investigating *MCPH1* function is of particular interest. Here, an overview of the key issues relating to the function of *MCPH1* in brain development and evolution will be reviewed.

## Autosomal Recessive Primary Microcephaly (MCPH)

Microcephaly is the clinical finding of a small brain, typically measured by head circumference (HC), compared to the population mean values of the age, sex, and ethnicity of the individual (Woods, 2004; Kaindl et al., 2010; Woods and Parker, 2013). HC, or more specifically occipito-frontal circumference (OFC) is commonly used as a surrogate measure of brain size (Woods et al., 2005); head size being a readily measurable approximation of brain size, and thus the terms microcephaly (small head) and microencephaly (small brain) are generally interchangeable (Gilmore and Walsh, 2013). An OFC of three standard deviations below the age- and sex-matched means (<−3 SD) is commonly accepted as a clinical definition of microcephaly (Woods and Parker, 2013). Furthermore, microcephaly is subdivided into primary and secondary microcephaly (Qazi and Reed, 1973). Primary microcephaly (microcephaly *vera*) is generally defined as being present at birth, with no obvious abnormalities other than gross brain size (Mochida and Walsh, 2001), and has a variety of genetic and non-genetic causes such as infections (Woods and Parker, 2013), prenatal radiation exposure (Plummer, 1952; Wood et al., 1967), and prenatal alcohol exposure (Ouellette et al., 1977; Spohr et al., 1993). Secondary microcephaly is generally defined as manifesting after birth (Woods and Parker, 2013). From the perspective of investigating brain development, the distinction between primary and secondary microcephaly is key, as primary microcephaly is likely to be related to neurogenesis, and secondary microcephaly may involve any of the downstream processes post-neurogenesis (Woods, 2004; Woods et al., 2005). Furthermore, microcephaly of genetic etiology, or autosomal recessive primary microcephaly, allows the detailed dissection of the relevant molecular mechanisms. The pathological defect in primary microcephaly is likely to fall temporally within the neurogenic interval and spatially within the neural progenitor cell compartment, making it ideal for the investigation of the regulation of brain size. However, in this context it is important to note that neuronal death due to alterations in diverse aspects of neuronal differentiation (including but not limited to neuronal migration and maturation) cannot be excluded, and may also account for the decrease in brain size seen in primary microcephaly. The main challenge in this regard is to determine how the alteration of these different processes at multiple stages of cortical development, ultimately impacts the final brain size.

Autosomal recessive primary microcephaly (MCPH) is a genetically heterogeneous condition (Mochida and Walsh, 2001), with 12 or more causative genes identified, all of which produce a clinically indistinguishable phenotype. The loci are numbered MCPH1 to MCPH12 (Kaindl, 2014), and the genes identified in this order are: *MCPH1* (Jackson et al., 1998, 2002), *WDR62* (Roberts et al., 1999; Nicholas et al., 2010), *CDK5RAP2* (Moynihan et al., 2000; Bond et al., 2005), *CASC5* (Jamieson et al., 1999; Genin et al., 2012), *ASPM* (Jamieson et al., 2000; Pattison et al., 2000; Bond et al., 2002), *CENPJ* (Leal et al., 2003; Bond et al., 2005), *STIL* (Kumar et al., 2009), *CEP135* (Hussain et al., 2012), *CEP152* (Guernsey et al., 2010), *ZNF335* (Yang et al., 2012), *PHC1* (Awad et al., 2013), and *CDK6* (Hussain et al., 2013). Mutations in *CEP*63 have also been identified as causing primary microcephaly (Sir et al., 2011). *ASPM* (*MCPH5*) is reported as being the most common locus for MCPH, with *WDR62* (*MCPH2*) being the second, followed by *MCPH1* and the other loci being rarer causes of MCPH (Roberts et al., 2002; Darvish et al., 2010; Nicholas et al., 2010; Sajid Hussain et al., 2013).

It has long been noted that the MCPH genes are all related to the mitotic spindle or centrosome (Bond and Woods, 2006; Bettencourt-Dias et al., 2011), and most MCPH proteins are located at the centrosome or spindle. However the more recently identified genes do not appear to be related to these functions, such as *PHC1* which plays a role in chromatin remodeling and DNA repair (Awad et al., 2013). It is interesting to note that MCPH1 traverses multiple functions related to: transcription activation (Yang et al., 2008), mitotic spindle and centrosome (Gruber et al., 2011), and DNA damage repair (Zhou et al., 2013), which raises a possible link between DNA repair and microcephaly. Although the hitherto known functions of the MCPH genes are linked to the cell cycle, the fact that the loss-of-function mutation of only one gene is sufficient to cause microcephaly excludes the possibility of functional redundancy. Nevertheless, such exclusion does not rule out genetic interactions between MCPH genes. Further investigations of the spatio-temporal expression patterns of these genes during cortical development and a detailed assessment of the neurodevelopmental defects occurring in MCPH patients and mouse model mutants will be necessary to answer the question of a crosstalk between MCPH genes, and the identity of a common centrosome- or spindle-related mechanism regulating brain size (Bond and Woods, 2006).

## Genetics of *MCPH1*

*MCPH1* was the first locus identified for MCPH, initially mapped to 8p22-pter (Jackson et al., 1998). The gene was later identified and the protein was named Microcephalin (Jackson et al., 2002). A feature unique to primary microcephaly caused by mutations in *MCPH1* are defects in chromosome condensation, specifically premature chromosome condensation (PCC) in early G2-phase, and delayed decondensation post-mitosis (Trimborn et al., 2006). This condition was originally named PCC syndrome (Neitzel et al., 2002); however mutations in *MCPH1* were later identified, and PCC and primary microcephaly caused by mutations in *MCPH1* were found to be allelic disorders (Trimborn et al., 2004). PCC is detected in cytogenetic preparations, as a high proportion of prophase-like cells and poor quality metaphase G-banding (Trimborn et al., 2004). PCC is a pathognomonic feature of *MCPH1*, being absent in all other mutations of MCPH genes. Another autosomal recessive condition characterized by microcephaly with additional craniofacial features and mitotic and chromosomal defects (Tommerup et al., 1993), was later found to also be caused by mutations in *MCPH1* (Farooq et al., 2010). *MCPH1* was also identified independently in a screen for transcriptional repressors of hTERT (catalytic subunit of human telomerase) and named *BRIT1*, BRCT-repeat inhibitor of hTERT expression (Lin and Elledge, 2003), which was later shown to be the same gene as *MCPH1* (Lin et al., 2005). Taken together, these molecular features of *MCPH1* collectively reveal that the gene is implicated in diverse processes that are crucial not only for proper brain development, but also for the maintenance of genome integrity (Liang et al., 2010).

## MCPH1 Protein Structure and Function

**Figure 1:**
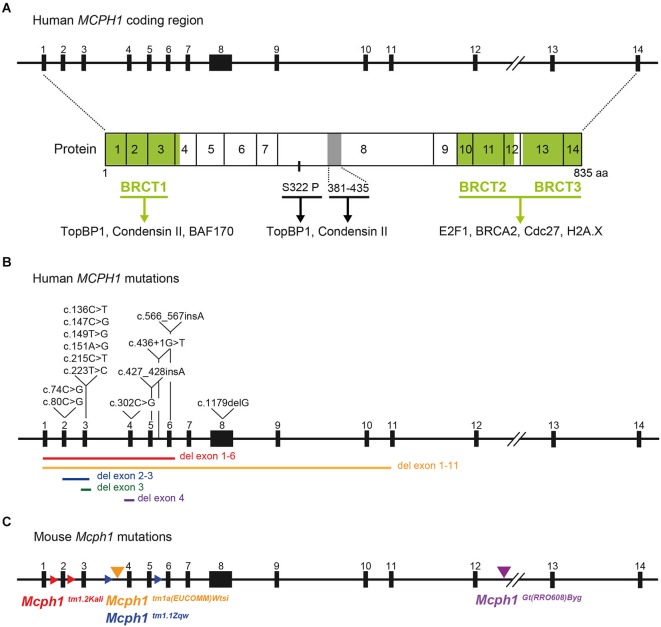
**Schematic of MCPH1 gene and protein domain structures and the positions of reported mutations in humans and mice. (A)** Schematic of *MCPH1* gene intron-exon structure (upper) and protein domain structures (lower), which are highly conserved between mouse and human. The *MCPH1* coding sequence includes 14 exons shown as black rectangles numbered from 1–14. The three BRCT domains are shaded in green. Factors interacting with the BRCT domains are indicated below each domain. A domain including residues 381–435 in exon 8 and interacting with condensin II is shaded gray, and the phosphorylation site Ser322 (S322P) important for TopBP1 recruitment is also shown. **(B)** Reported mutations in *MCPH1* causing primary microcephaly are indicated on the gene schematic (see Table 1; Figure 2 for amino acid changes). The extent of the deletions is indicated as colored bars below the gene structure. **(C)** Schematic of the four reported *Mcph1* mutations in mice (see also Table 2). For targeted deletions, the triangles overlaid on the intron flanks the exons that were targeted for deletion, with the allele name indicated by the same color below. For the gene trap mutation and knockout-first allele, the site of the insertion is indicated by a triangle above the intron, with the corresponding allele name in the same color.

**Figure 2:**
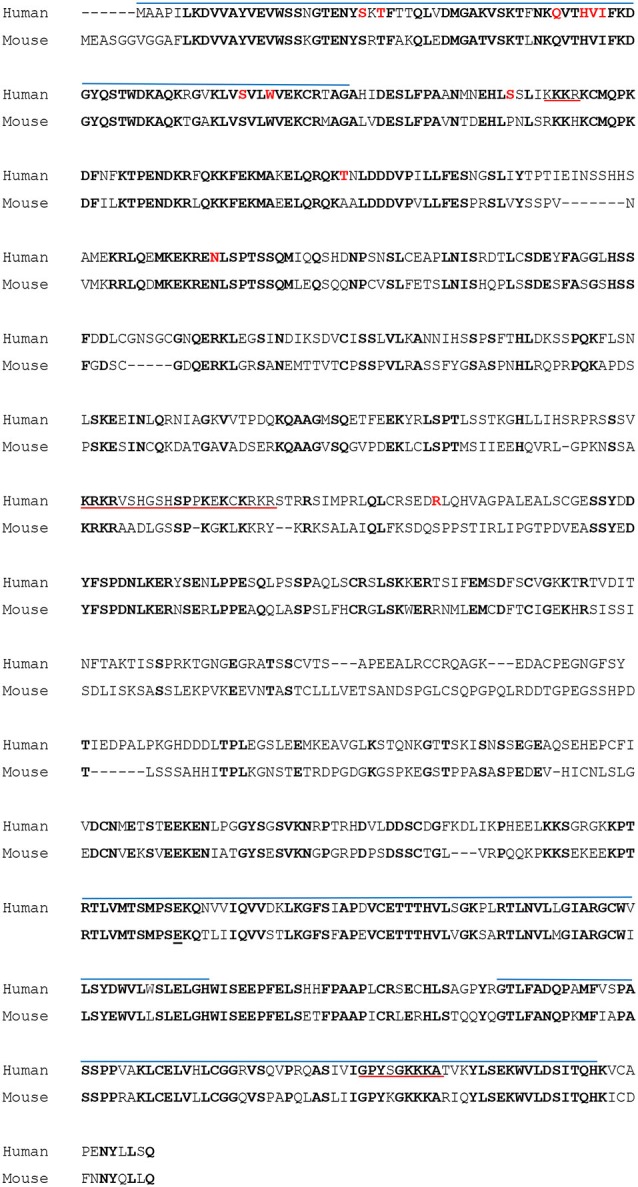
**ClustalX alignment of human MCPH1 and mouse Mcph1 proteins**. Conserved amino acids are shown in bold characters. Blue upper lines indicate BRCT domains, and red underlines indicate nuclear localization signals (Gavvovidis et al., 2012). Amino acid residues mutated in microcephaly (either nonsense or missense; see Table 1; Figure 1) are in red.

In human and mouse (*Mus musculus*), the *MCPH1* coding sequence contains 14 exons, distributed across 200 kb of genomic DNA. The protein contains three BRCT (**BR**CA1 **C**-**T**erminal) domains (Figures 1, 2), which were first described in BRCA proteins and mediate protein-protein interactions (Koonin et al., 1996; Huyton et al., 2000). One domain is located at the N-terminal side of the MCPH1 protein (BRCT1), and a tandem of two domains spans the C-terminal sequence (BRCT2/3). These domains are predicted to be crucial for function (Jeffers et al., 2008) and may be differentially involved in the diverse roles of MCPH1 by mediating interactions with distinct partners. In line with this notion, repeats of BRCT domains, such as BRCT2/3, have been shown to preferentially interact with phosphorylated residues (Woods et al., 2012).

**Table 1:** **Reported mutations in *MCPH1* causing autosomal recessive primary microcephaly**.

Mutation	Protein	Exon	HC	Reference
del exon 1–6	?	1–6	−3 SD	Garshasbi et al. (2006), Darvish et al. (2010)
del exon 1–11	?	1–11	−3 SD (birth)	Pfau et al. (2013)
			<–5 SD (10 m)
c.74C>G	p.Ser25Ter	2	−5 to −10 SD	Jackson et al. (2002)
c.80C>G	p.Thr27Arg	2	−2.4 SD (birth)	Trimborn et al. (2005)
			−3 SD (6 yr)
del exon 2–3	?	2–3	−6 to −8 SD	Darvish et al. (2010)
del exon 3	?	3	−6 to −10 SD	Darvish et al. (2010)
c.136C>T	p.Gln46Ter	3	<–4 SD	Hosseini et al. (2012)
c.147C>G	p.His49Gln	3	−7 to −9 SD	Darvish et al. (2010)
c.149T>G; c.151A>G	p.Val50Gly; p.Ile51Val	3	<0.4th centile	Leung et al. (2011)
c.215C>T	p.Ser72Leu	3	−6 to −7 SD	Darvish et al. (2010), Ghani-Kakhki et al. (2012)
			−3.5 SD (birth)
c.223T>C	p.Trp75Arg	3	−5.7 SD (birth)	Ghani-Kakhki et al. (2012)
del exon 4	?	4	−10 to −11 SD	Darvish et al. (2010)
c.302C>G	p.Ser101Ter	4	?	Farooq et al. (2010)
c.427_428insA	p.Thr143Asnfs	5	−8 to −10 SD	Trimborn et al. (2004)
c.436 + 1G>T	Splice mut.	Intr. 5	−9 SD	Darvish et al. (2010)
c.566_567insA	p.Asn189fs	6	−6 SD	Darvish et al. (2010)
c.1179delG	p.Arg393Serfs	8	−8 to −10 SD	Sajid Hussain et al. (2013)

*The genotype, predicted effect on protein (“?” if unknown or not described), the exon containing the mutation, standard deviation (SD) of the head circumference (HC), and the reference are shown. All mutations are homozygous*.

**Table 2:** **Summary of mouse models of human *MCPH1***.

Genotype/gene product	Phenotype	Reference
***Mcph1^Gt(RRO608)Byg^***	Body weight normal	Trimborn et al. (2010)
Hypomorphic mutation	Brain/body weight normal
Gene trap between exons 12–13	Brain volume normal (MRI)
Predicted to lack C-term 97aa	Defective chromosome condensation
Residual *wt* transcript detected	Shortened lifespan; fertility normal

***Mcph1^tm1.2Kali^***	Growth retardation	Liang et al. (2010)
Targeted deletion of exon 2	IR hypersensitivity
Null mutation	Smaller testis and ovary; infertile
	Spermatogenesis defects
	No ovarian follicles
	*Hom* born at lower than Mendelian ratio

***Mcph1^tm1.1Zqw^***	Brain weight reduced (~20%)	Gruber et al. (2011), Zhou et al. (2013)
Targeted deletion of exon 4–5	Growth retardation (~20%)
Null mutation	Brain/body weight normal
	Reduction of neocortex in radial and lateral dimensions; cerebellum normal
	Neural progenitor apoptosis and increased cell cycle exit; deviation of progenitor mitotic cleavage plane
	Smaller testis and ovary; infertile

***Mcph1^tm1a(EUCOMM)Wtsi^***	Brain weight reduced (~15%)	Chen et al. (2013)
Hypomorphic mutation	Growth normal
Knockout-first allele	*Hom* born at lower than Mendelian ratio
Insertion between exons 3–4	Infertile
Residual *wt* transcript detected	Hearing impairment

*Genotypes of mouse Mcph1 mutations, the phenotypes of the mice, and the references are shown. Allele names obtained from Mouse Genome Informatics (MGI:2443308). MRI, magnetic resonance imaging; IR, ionizing radiation; Hom, homozygous mutant; wt, wildtype*.

A number of studies have reported on MCPH1 interaction partners and differential functions of the N-terminal BRCT1 domain, and the more C-terminal BRCT2/3 domains (Figure 1). Two regions of human MCPH1: an N-terminal fragment containing BRCT1 (residues 1–195), and a central fragment (residues 381–435) interact with different subunits of condensin II to regulate chromosome condensation (Yamashita et al., 2011). The N-terminal sequence (residues 1–48) was shown to be necessary to recruit BAF170, a component of the chromatin remodeling complex SWI/SNF, to relax chromatin for DNA repair (Peng et al., 2009). This suggests a possible role of MCPH1 in chromatin conformation. BRCT2/3 domains have been shown to bind to E2F1 to form a complex able to transactivate BRCA1 and CHK1 (Yang et al., 2008). Interactions between MCPH1 BRCT2/3 domains and Cdc27, a subunit of the anaphase-promoting complex (Singh et al., 2012b), and a phosphorylated domain of H2A.X have also been reported (Singh et al., 2012a). On the other hand, a truncated MCPH1 protein lacking BRCT2/3, is able to complement the defective chromosome condensation in human MCPH1-deficient cells (Gavvovidis et al., 2012), indicating that these domains are dispensable for this function. Molecular interactions between MCPH1 and partners may likely be regulated by post-translational modifications. In line with this notion, Ser322 phosphorylation of MCPH1 by ATR is required for TopBP1 recruitment and sustained ATR signaling for DNA repair (Zhang et al., 2014). Further mechanistic dissection of the functions of BRCT1 and BRCT2/3 domains will allow a better definition of the different molecular and biochemical mechanisms impaired by *MCPH1* mutations.

## Human *MCPH1* Mutations

A large number of homozygous mutations in *MCPH1* causing primary microcephaly have been identified (Table 1; Figures 1, 2). These include large deletions of exons (Garshasbi et al., 2006; Darvish et al., 2010; Pfau et al., 2013), single base-pair insertions and deletions (Trimborn et al., 2004; Darvish et al., 2010; Sajid Hussain et al., 2013), nonsense mutations (Jackson et al., 2002; Farooq et al., 2010; Hosseini et al., 2012), missense mutations (Trimborn et al., 2005; Darvish et al., 2010; Leung et al., 2011; Ghani-Kakhki et al., 2012), and one splice site mutation (Darvish et al., 2010). Interestingly, all missense mutations identified so far are located in exons 2 or 3 (Table 1; Figure 1), which is within the N-terminal BRCT domain (BRCT1; see previous section on MCPH1 protein). The missense mutations all lead to non-conservative changes in an amino acid residue: threonine to arginine (c.80C>G, p.Thr27Arg) which is polar uncharged to positively charged (Trimborn et al., 2005), histidine to glutamine (c.147C>G, p.His49Gln) which is positively charged to polar uncharged (Darvish et al., 2010), serine to leucine (c.215C>T, p.Ser72Leu) which is polar uncharged to non-polar (Darvish et al., 2010; Ghani-Kakhki et al., 2012), and tryptophan to arginine (c.223T>C, p.Trp75Arg) which is non-polar aromatic to positively charged (Ghani-Kakhki et al., 2012). These mutated residues are highly conserved during evolution, Thr27 being conserved in orthologs of *MCPH1* in mammals and amphibians (Trimborn et al., 2005). Ser72 and Trp75 are conserved in all vertebrates and *Drosophila*, and these two residues are also conserved in BRCT domains of *BRCA1* (Ghani-Kakhki et al., 2012). In one case a homozygous double mutation in consecutive codons (c.149T>G, c.151A>G; p.Val50Gly, p.Ile51Val) was found, which leads to conservative residue changes (both non-polar to non-polar); however still within the N-terminal BRCT domain. Molecular and biochemical studies of full-length MCPH1 proteins harboring these various amino acid changes may be a powerful tool in correlating *MCPH1* genotype and molecular function with phenotype.

As previously noted for *ASPM* (Nicholas et al., 2009), correlations between mutation and phenotype, such as the severity of microcephaly, could not be established so far (Table 1). One notable exception is the p.Thr27Arg missense mutation, which shows only a mild microcephaly (−2.4 SD at birth), and the fraction of prophase-like cells being only marginally higher than controls (Trimborn et al., 2005; Ghani-Kakhki et al., 2012). Meta-analysis of genotype and phenotype correlations are hampered however by the rarity of patients with *MCPH1* mutations, and by the fact that the HC data is reported at birth in some cases (Trimborn et al., 2005) but often at older ages for others, and often only a range of HCs are shown for multiple individuals within a family (Darvish et al., 2010). Similarly, PCC is often reported qualitatively (Pfau et al., 2013), without a careful quantification of prophase-like cells as seen in other studies (Ghani-Kakhki et al., 2012). Molecular studies comparing the activities of the mutant proteins may aid in answering some of these questions (Leung et al., 2011), and will provide powerful clues in understanding the mechanisms of MCPH1.

## Mouse Models of *Mcph1* Deficiency: Microcephaly Phenotype

A number of mouse models for *Mcph1* loss-of-function have been reported (Table 2; Figure 1). These mutant mouse lines have been successful in recapitulating the features of primary microcephaly and PCC caused by mutations in *MCPH1* in humans. The first study demonstrating a microcephaly phenotype in a *Mcph1* null mutant mouse model (Gruber et al., 2011) was generated by targeted deletion of *Mcph1* exon 4–5 (*Mcph1^tm1.1Zqw^*). A follow-up study of this mouse model was reported by the same group (Zhou et al., 2013). In homozygous *Mcph1^tm1.1Zqw^* mutant mice, the size of the newborn mouse brain was visibly smaller, and brain weight was also reduced, fulfilling the criteria of human primary microcephaly at a gross anatomical level. Histological examination of the newborn brains revealed an approximate 20% reduction in both the radial thickness and the lateral extent of the neocortex. The *Mcph1* mutant brain at embryonic day (E) 13.5 was reported as reduced, and the cortical wall was thinner at E15.5 (Gruber et al., 2011), implying an early defect. Interestingly, in adult mutant mice, the anterior portion of the brain (defined in this case as the olfactory bulb, cerebrum, and thalamus) showed a more significant reduction compared to the remaining posterior portion. Moreover, histological examination of the cerebellum at post-natal day (P) 21 was reportedly normal suggesting that *MCPH1* may not be required for the development of the cerebellar cortex (Zhou et al., 2013).

Importantly, *MCPH1* mutant mice were also reported to have an approximate 20% reduction in body weight (at P0, P21, and P180; i.e., at birth, weaning, and adult) and the proportion of brain weight to body weight showed no significant difference compared to controls (Zhou et al., 2013). Short stature is a variable feature of human *MCPH1* and is seen in some patients (Trimborn et al., 2004). However, a proportionate reduction in both brain and body weight, raises questions of the specificity of *Mcph1* as a sole brain size regulator, at least in the mouse model. In another mouse model, a *Mcph1* null mutant generated by a targeted deletion of exon 2 (*Mcph1^tm1.2Kali^*), growth retardation was also found, also showing an approximate 20% reduction in weight; however brain weights were not reported in this study (Liang et al., 2010). The answer to the question of a specific effect of Mcph1 on brain size, rather than an effect on overall body size, will require the analysis of mouse conditional mutants, with specific inactivation in the brain or in the neocortex. In contrast, reported mouse models of *ASPM* show a specific reduction in brain weight, with no or minimal reduction in body weight (Pulvers et al., 2010; Fujimori et al., 2014).

Two other mouse models of *Mcph1* have been reported, one generated from gene trap ES cells (Skarnes et al., 2004), and another harboring a knockout-first allele (Skarnes et al., 2011). The model containing a gene trap vector between exons 12–13 (*Mcph1^Gt(RRO608)Byg^*), which leads to a protein lacking only the most C-terminal BRCT3 domain, showed normal body and brain weight, however did exhibit the PCC phenotype (Trimborn et al., 2010). The knockout-first allele mouse model with an insertion between exons 3–4 (*Mcph1^tm1a(EUCOMM)Wtsi^*) showed brain weight reduction (~15%); however there was no evidence of growth retardation (Chen et al., 2013). Both of these *Mcph1* models are hypomorphic mutants with detectable residual wild-type transcript, and it is thus difficult to interpret these phenotypes and compare them to the other *Mcph1* null mutants and human *MCPH1* patients.

## Mouse Models of *Mcph1* Deficiency: Non-Brain Phenotypes

A number of other phenotypes have been reported in the *Mcph1* mutant mice (Table 2). Infertility has been observed in the two null mutants (Liang et al., 2010; Gruber et al., 2011; Zhou et al., 2013) and one of the hypomorphic mutants (Chen et al., 2013). Phenotypes were observed in both spermatogenesis and in the ovary (Liang et al., 2010). Germline phenotypes have also been reported for mouse models of *ASPM* (Pulvers et al., 2010; Fujimori et al., 2014). This raises the important question of whether infertility, or testicular or ovarian atrophy, is seen in human primary microcephaly. So far no cases have been reported and the relationship between primary microcephaly and infertility in humans remains an interesting question, as it may provide clues to the mechanism of the gene, and also raises the possibility that the function of MCPH1 in the germline may have been the target of positive selection in the primate lineage (Woods et al., 2006; Dobson-Stone et al., 2007; Timpson et al., 2007).

In one of the *Mcph1* hypomorphic mutants (*Mcph1^Gt(RRO608)Byg^*), homozygous mice exhibited a shortened lifespan, specifically a significant difference in survival after 65 weeks of age (Trimborn et al., 2010). This observation of decreased overall survival in *Mcph1* homozygous mutants was replicated in the *Mcph1^tm1.2Kali^* null mutant, which manifests an increased cancer susceptibility (Liang et al., 2014). This difference in survival was not reported in the other mutants (Gruber et al., 2011; Chen et al., 2013; Zhou et al., 2013); however as the difference becomes apparent when mice are older, a shortened lifespan may have been overlooked. This raises the question of whether patients with primary microcephaly exhibit a shortened lifespan, independent of other comorbidities that may be exacerbated by the associated mental retardation. Epidemiological studies may be difficult due to the rarity of MCPH. However, one post-mortem case report of a 77-year old with primary microcephaly exists (McCreary et al., 1996) and another study reports on MCPH patients ranging to their 70 s (Darvish et al., 2010) indicating that the condition is compatible with normal lifespan.

Another interesting phenotype of *Mcph1* mutant mice reported in two studies (Liang et al., 2010; Chen et al., 2013) is a significantly reduced proportion of homozygous mice (10~15% vs. the expected normal 25% Mendelian ratio) born from intercrossing of heterozygous *Mcph1* mutant mice. This indicates the possibility of an embryonic lethal phenotype of varying penetrance caused by homozygous mutations in *Mcph1*.

## MCPH1 and Neurogenesis

The vastly complex cerebral cortex has its developmental origin in the germinal zone, or ventricular zone (VZ), of the dorsal telencephalon of the neural tube (Götz and Huttner, 2005). The VZ is composed of neuroepithelial cells, which line along the ventricles of the embryonic brain vesicles and spinal cord, and serve as the primary progenitor cells of all neural cells of the central nervous system. Neuroepithelial cells exhibit typical epithelial cell characteristics, such as apical-basal polarity (Chenn et al., 1998), and its nuclei undergo a unique movement in the apical-basal axis correlated with the cell cycle, termed interkinetic nuclear migration (Sauer, 1935; Taverna and Huttner, 2010; Kosodo, 2012), which gives the tissue a pseudostratified histological appearance, and thus its classification as a pseudostratified epithelium. Neuroepithelial cells, or neural progenitor cells, initially proliferate to expand the progenitor pool, and later commence differentiative divisions into downstream progenitors and neurons (Paridaen and Huttner, 2014), the process of neurogenesis.

In cortical development, the lateral dimension of the laminar structure is largely dictated by the expansion of neural progenitor cells or proliferative units, and the radial dimension is a result of the extent of neurogenesis subsequent to progenitor proliferation within an ontogenetic column or radial-unit (Rakic, 1988, 1995, 2009). This framework allows the dissection of cortical phenotypes, where a reduction in the lateral dimension is likely a deficit in the initial progenitor pool and its subsequent proliferative expansion, and a reduction in the radial dimension may be caused by a reduced capacity for neurogenesis, shortening of the neurogenic interval, or neuronal loss. Conceptually, an analysis distinguishing the radial and lateral dimensions is useful in phenotypic dissection of mutant mice to further clarify their respective impact on the final neurogenic outcome.

*Mcph1* inactivation has an impact on both lateral and radial dimensions (Gruber et al., 2011; Zhou et al., 2013). These characteristics have been related to a loss of progenitors and to a specific decrease in upper layer neuron output, possibly due to premature progenitor exhaustion. They appear to result from the deficiency of two distinct *Mcph1* functions: the control of the centrosome cycle through the Chk1-Cdc25 pathway, and DNA damage repair. The relative contribution of each deficiency in this progenitor loss has not been addressed and will require further studies. Likewise, it will be interesting to address whether the aforementioned participation of Mcph1 in chromatin conformation during DNA repair could also be important for chromatin conformation with regards to progenitor fate determination during neurogenesis. In line with this notion, competitive interactions between BAF170 and BAF155 with Pax6 have been shown to play an important role in the choice between cell cycle maintenance vs. differentiation (Tuoc et al., 2013). Interestingly, mutations of several BAF genes, including BAF170 and BAF155, have been reported to be involved in brain disorders (Ronan et al., 2013), and it will be interesting to further explore how interactions between BAF and MCPH1 proteins impact brain size and function.

The full interpretation of the defects reported for *Mcph1* mutant mice is hampered by the lack of detailed information on the pattern of Mcph1 expression during cortical development, such as the temporal dynamics of gene activation, the cell types expressing the gene and the sub-cellular localization of the Mcph1 protein. It remains thus difficult to establish a direct link between the described phenotypes and gene function. Moreover, gaining insight into the spatio-temporal mode of *MCPH1* expression in mouse and human will also allow the delineation of how the function of the gene may have diverged between both species. In particular, it will be interesting to determine if *MCPH1*, along with other MCPH genes, are expressed in the OSVZ (outer subventricular zone) progenitors (Smart et al., 2002), and eventually how the MCPH genes may impact the proliferation rate and division mode of these progenitors, which are considered to be involved in the expansion of the surface area of the neocortex (Lui et al., 2011; Betizeau et al., 2013; Sun and Hevner, 2014).

## MCPH1 and Brain Evolution

Ever since the identification of *MCPH* genes, there has been intense interest in their possible roles in the evolution of brain size (Evans et al., 2004; Wang and Su, 2004; Ponting and Jackson, 2005; Woods et al., 2005). Microcepaly (primary microcephaly, or microcephaly *vera*) has long been the subject of evolutionary interest, and is commonly described in the literature as an atavistic (or “throwback”) condition, a reversion to an ancestral form (Mochida and Walsh, 2001; Gilbert et al., 2005; Ponting and Jackson, 2005; Vallender et al., 2008). In fact, microcephaly was already proposed in the mid-19th centrury by Carl Vogt to be the reappearance of an ancestral primate (Komai et al., 1955; Richardson, 2011). Much of the intrigue and fascination of research into *MCPH* genes has been fueled by the remarkable phenotype of primary microcephay: a significant and specific reduction in brain size with the absence of other neurological and non-neurological abnormalities (Woods et al., 2005). However, as little is known about the actual disturbance in cortical structure and cytoarchitecture in *MCPH*, and since the neuroanatomy of ancestral primates and hominids may forever remain in the realm of speculation and extrapolation from the comparison of extant species (Holloway, 1968; Rilling and Insel, 1999; Falk et al., 2000), parallels between the pathological condition of microcephaly, and the evolutionary change in primate brain size must be made with caution. Moreover, *MCPH* cannot be described as “atavistic” in genetic terms, as all mutations identifyed so far, whether they result in loss-of-function or the truncated MCPH1 protein has a toxic effect, are mutations predicted to cause gene dysfunction, not a reversal to an ancestral sequence (Gilbert et al., 2005). However, the MCPH phenotype does provide insight into a cell biological mechanism of brain size regulation, which may indeed have been involved in primate brain evolution (Bond and Woods, 2006; Fish et al., 2008; Thornton and Woods, 2009; Sun and Hevner, 2014).

Intriguinly, *Mcph1* loss of function in mice primarily affects the number of upper-layer neurons (Zhou et al., 2013), which are very important for intra-cortical connections and are involved in higher cognitive functions. The output of these neurons has increased during evolution correlating with increasing complexity of cognitive functions, as observed in humans and primates (DeFelipe et al., 2002; Molnar et al., 2006; Fame et al., 2011). The participation of *MCPH* genes in the development of upper-layer neurons (Lizarraga et al., 2010; Yang et al., 2012; Zhou et al., 2013) may thus represent a major clue for understanding the evolution of brain size and function.

Regarding studies on the evolution of *MCPH* genes, interest has generally centered on (i) the possibility of genetic variations in these genes being directly involved in brain size regulation during primate and human evolution; or (ii) through the analysis of the function of these genes a molecular mechanism or pathway regulating brain size may be identified.

### MCPH1: Positive Selection and Polymorphisms

With regards to variations in *MCPH1*, studies have focussed on either the analysis of positive (or Darwinian) selection of *MCPH1* in extant primate species, or through the analysis of polymophisms in human populations. Positive selection in the context of protein evolution can be studied by the analysis of the ratio of non-synonymous (*K_a_*) to synonymous (*K_s_*) changes in DNA sequence (Yang and Bielawski, 2000; Hurst, 2002), and has commonly been used in investigating the link between brain development and evolution (Dorus et al., 2004; Gilbert et al., 2005). Briefly, since synonymous mutations in codons do not change amino acid sequence and therefore do not alter the biochemical properties of the protein, they are assumed to be selectively neutral and reflect the neutral mutation rate. Non-synonymous mutations on the other hand, alters the amino acid sequence which may in turn alter protein function, which more commonly would lead to gene dysfunction and an evolutionary disadvantage, and rarely may confer a gain-of-function or an evolutionary advantage (Woods et al., 2005). Therefore a non-synonymous/synonymous substitution ratio (*K_a_/K_s_* or *dN/dS*) of >1 can be interpreted as evidence for positive selection. Utilizing these methods, *MCPH1* was found to exhibit positive selection in the primate lineage, specifically from the common ancestor of great apes and humans (Evans et al., 2004; Wang and Su, 2004). Another study which investigated four microcephaly genes across 21 species of anthropoid primates identified positive selection correlating with neonatal and adult brain size for *ASPM* and *CDK5RAP2*, but interestingly not for *MCPH1* and *CENPJ* (Montgomery et al., 2011). Positive selection of *MCPH1* was also identified in cetaceans, which was however also not associated with variations in brain size (McGowen et al., 2011). Examination of positive selection of microcephaly genes across 33 eutherian mammal species revealed signs of positive selection of *MCPH1* across non-primate mammals, however *MCPH1* did not correlate with neonatal brain size (Montgomery and Mundy, 2014). How *MCPH1* may be involved in brain size regulation more broadly across vertebrates and whether variations show any correlations between gyrencephalic vs. non-gyrencephalic species remains an interesting question.

A large number of non-pathogenetic mutations or polymorphisms in *MCPH1* are known, particularly in exon 8 and 13 (Scala et al., 2010). One of which, c.940G>C (p.Asp314His, non-conservative missense from negatively to positively charged, exon 8; rs930557), has received much attention. This polymorphism which is diagnostic for a haplotype with the derived C allele, designated as haplogroup D, has a relatively young coalescence age (i.e., time to single ancestral copy) of 37,000 years, however with a high population frequency worldwide, indicating strong positive selection among anatomically modern humans (Evans et al., 2005). Further analysis on the origin of haplogroup D indicated its divergence 1.1 million years ago from the human lineage and subsequent introgression of this derived allele into human populations 37,000 years ago, possibly due to interbreeding between humans and an archaic *homo* species, which was speculated as Neanderthals (Evans et al., 2006). Although Neanderthals were suggested as the possible archaic *homo* (Hawks et al., 2008), subsequent sequencing of *MCPH1* of Neanderthals revealed the non-D (ancenstral) haplotype (Green et al., 2010; Lari et al., 2010).

A large number of studies have attempted at identifying associations between *MCPH1* haplogroup D and a variety of brain-related phenotypes. In short, no associations have been identified with regards to HC, brain size by MRI, IQ or mental retardation (Woods et al., 2006; Dobson-Stone et al., 2007; Mekel-Bobrov et al., 2007; Rushton et al., 2007; Timpson et al., 2007; Bates et al., 2008; Maghirang-Rodriguez et al., 2009). One study identified an association with the population freqency of haplogroup D and linguistic tone (Dediu and Ladd, 2007), although a later study showed that the derived allele is not associated with lexical tone perception (Wong et al., 2012).

The derived allele of another* MCPH1* polymorphism, c.2282T>C (p.Val761Ala, conservative missense from non-polar to non-polar, exon 13; rs1057090), was associated with an increase in cranial volume in Chinese males, but not females (Wang et al., 2008). Non-exonic common variants in *MCPH1* have been associated with brain size and cortical surface area in females (Rimol et al., 2010), and another study has investigated *MCPH* genes and their association with sexual dimorphism in brain size in primates (Montgomery and Mundy, 2013). Mechanistically, how *MCPH1* may contribute to sexually-dimorphic brain phenotypes remains unclear.

Key questions remain as to whether *MCPH1* did play a role in the evolution of brain size in the primate lineage, and whether common variants of *MCPH1* in human populations today are associated with any structural or functional brain phenotype. In light of the phenotypic data from mouse models (Liang et al., 2010; Trimborn et al., 2010; Gruber et al., 2011; Chen et al., 2013; Zhou et al., 2013) which implicate *MCPH1* in a number of other non-nervous systems, notably the germline, positive selection in primates and humans may not be due to adaptive changes in brain size or function (Woods et al., 2006; Dobson-Stone et al., 2007; Timpson et al., 2007). In this context it is interesting to note that a large scale study of human and chimpanzee orthologs for evidence of positive selection revealed a large number of genes involved in tumor suppression, apoptosis, and spermatogenesis (Nielsen et al., 2005); functions where *MCPH1* may play a major role. Interestingly, two *MCPH1* polymorphisms have been associated with breast cancer risk (Jo et al., 2013), which further stresses the need of examining non-nervous system phenotypes in the context of *MCPH1* evolution and also in primary microcephaly patients.

### MCPH1 Evolution: From a Cell Biological Perspective

Cortical development in rodents and primates share many features, however there are a number of important differences relevant for brain size evolution. A key difference and one that is of relevance to *MCPH1*, is the mechanisms which regulate the production of progenitors in the OSVZ, the germinal compartment which has enlarged strikingly in primates and humans and is considered as the seat of the evolutionary expansion of neocortical surface area (Smart et al., 2002; Lui et al., 2011; Sun and Hevner, 2014). The analysis and comparison of these differences in progenitor cell types and lineage relationships, germinal layer cytoarchitecture, and cell biological mechanisms will help in constructing a model of mammalian brain evolution from a developmental and cell biological perspective (Fish et al., 2008; Rakic, 2009; Fietz and Huttner, 2011; Lui et al., 2011; Sun and Hevner, 2014). Another clue recently emerged is the importance of DNA repair pathways, revealed by a preferential effect of mutations of genes implicated in such pathways, including *MCPH1*, on neural progenitors (Gilmore and Walsh, 2013). This suggests the existence of a specific cross-talk between DNA repair pathways and primary cell cycle functions in these progenitors, which might have become more critical during evolution. Integrating these molecular findings with genetics and evolutionary biology will be a powerful approach in investigating brain size evolution (Enard, 2014).

With regards to *MCPH1*, some studies have taken this approach in investigating its function in brain size evolution. One study identified an E2F1 binding motif in the *MCPH1* promoter region, which is specific to primates and absent in mice and other vetebrates (Shi and Su, 2012). E2F1 is a transcription factor regulating genes involved in cell cycle and apoptosis (Ginsberg, 2002), and interestingly MCPH1 is involved in transcriptional regulation of several DNA repair, checkpoint and apoptosis genes, via interaction with E2F1 (Yang et al., 2008). Another study performed a cell line assay comparing human and rhesus macaque MCPH1 protein and its affects on down-stream gene expression, and found that human-specific amino acid changes in MCPH1 led to differences in expression of three downstream genes involved in cell cycle regulation and apoptosis (Shi et al., 2013). These studies go beyond correlating genetic changes with brain size, and attempt to experimentally test the hypothesis that *MCPH1* is an important gene in brain size evolution. Futher approaches may be to generate humanized and primatized mice expressing human and other primate *MCPH1* (Pulvers et al., 2010), or the use of cerebral organoids (Lancaster et al., 2013), an *in vitro* model of human cortical development, where microcephaly-causing mutations in humans and primate-specific variants in *MCPH1* can be investigated in detail in a system amenable to experimentation (Enard, 2014). Gene expression profiling studies aimed at identifying pathways dependent on *MCPH1* in mouse and human, as well as the characterization of molecular partners for both the mouse and human proteins, will provide major clues on the molecular mechanisms involving MCPH1 and its role in the evolution of brain size. Much may be learnt regarding the role of MCPH genes in brain size evolution, from constructing developmental and cell biological tools for analyzing evolutionary questions.

*MCPH1* is well-positioned as a candidate gene for understanding the mechanisms of brain size evolution, as it is related not only to the other primary microcephaly genes with regards to its phenotype, but also harbors the same BRCT domains as *BRCA1*, which has also been shown to be important for brain development (Pulvers and Huttner, 2009; Pao et al., 2014) and shows positive selection in the primate linage (Huttley et al., 2000; Lou et al., 2014).

## Conclusions

Advances in medical genetics have greatly enhanced our understanding of the origins of many brain and nervous system development disorders; microcephaly in particular. Nevertheless, the molecular and cellular processes underlying such disorders remain poorly understood, and gaining insight into the pathological mechanisms has remained as a major challenge in developmental neurobiology. In this respect, microcephaly of genetic etiology represents a valuable context for the study of the mechanisms that control the final neurogenic output, and by extension to animal models, to assess how these mechanisms have been adjusted and modulated during evolution along with the remarkable expansion of brain size. So far, interests have focused mainly on aspects related to cell division and proliferation; however the pathological mechanisms associated with microcephaly may prove to be more complex and multifactorial. In line with this notion, *MCPH1* appears to assume multifaceted functions, including but not limited to: brain development (Jackson et al., 1998, 2002), DNA damage repair (Xu et al., 2004; Lin et al., 2005; Peng et al., 2009), chromosome condensation (Neitzel et al., 2002; Trimborn et al., 2004; Yamashita et al., 2011), cancer (Chaplet et al., 2006; Rai et al., 2006; Richardson et al., 2011), and germline function (Liang et al., 2010), as reviewed here and elsewhere (Venkatesh and Suresh, 2014). Further comparative expression and functional studies in different species, including primates, will prove to be highly informative in further delineating the molecular and genetic networks controlled by *MCPH1*, and how they may have been tuned or co-opted to participate in the expansion of brain size. Such progress in the understanding of fundamental developmental mechanisms of the brain is expected to have a valuable impact not only in the understanding of clinical conditions such as microcephaly, but also to answer one of the most enduring questions in biology: *the evolution of brain size*.

## Conflict of Interest Statement

The authors declare that the research was conducted in the absence of any commercial or financial relationships that could be construed as a potential conflict of interest.
